# Histone Lysine Methylation in TGF-*β*1 Mediated p21 Gene Expression in Rat Mesangial Cells

**DOI:** 10.1155/2016/6927234

**Published:** 2016-05-09

**Authors:** Qiaoyan Guo, Xiaoxia Li, Hongbo Han, Chaoyuan Li, Shujun Liu, Wenhui Gao, Guangdong Sun

**Affiliations:** ^1^Department of Nephrology, 2nd Hospital of Jilin University, Changchun 130041, China; ^2^Department of Endocrinology, 208th Hospital of Chinese PLA, Changchun 130062, China; ^3^Department of Neonatology, 2nd Hospital of Jilin University, Changchun 130041, China

## Abstract

Transforming growth factor beta1- (TGF-*β*1-) induced p21-dependent mesangial cell (MC) hypertrophy plays a key role in the pathogenesis of chronic renal diseases including diabetic nephropathy (DN). Increasing evidence demonstrated the role of posttranscriptional modifications (PTMs) in the event; however, the precise regulatory mechanism of histone lysine methylation remains largely unknown. Here, we examined the roles of both histone H3 lysine 4 and lysine 9 methylations (H3K4me/H3K9me) in TGF-*β*1 induced p21 gene expression in rat mesangial cells (RMCs). Our results indicated that TGF-*β*1 upregulated the expression of p21 gene in RMCs, which was positively correlated with the increased chromatin marks associated with active genes (H3K4me1/H3K4me2/H3K4me3) and negatively correlated with the decreased levels of repressive marks (H3K9me2/H3K9me3) at p21 gene promoter. TGF-*β*1 also elevated the recruitment of the H3K4 methyltransferase (HMT) SET7/9 to the p21 gene promoter. SET7/9 gene silencing with small interfering RNAs (siRNAs) significantly abolished the TGF-*β*1 induced p21 gene expression. Taken together, these results reveal the key role of histone H3Kme in TGF-*β*1 mediated p21 gene expression in RMC, partly through HMT SET7/9 occupancy, suggesting H3Kme and SET7/9 may be potential renoprotective agents in managing chronic renal diseases.

## 1. Introduction

Glomerular diseases are the leading causes of chronic kidney disease (CKD) and end-stage renal disease (ESRD) worldwide; several renal cells including mesangial cells (MC), podocytes, epithelial cells, and endothelial cells are involved in the pathogenesis. MCs' response to injury includes apoptosis, proliferation, and hypertrophy, and MC hypertrophy is a hallmark of IgA nephropathy (IgAN), membranoprolifrative glomerulonephritis (MPGN), lupus nephritis (LN), and DN [1–4]. A lot of studies have shown the key role of cyclin-dependent kinase inhibitor (CDKI) such as p21 and P27 in MC hypertrophy [5–7]; attenuation of p21 by antisense oligodeoxynucleotide (ODN) could decrease MC hypertrophy induced by hyperglycemia and IGF-1 [8]; as a result diabetic p21 −/− mice did not develop glomerular hypertrophy; all data suggest p21 may be necessary for glomerular hypertrophy mediated by TGF-*β*1 in chronic renal diseases including DN [5, 9].

TGF-*β*1 has been implicated in initial glomerular hypertrophy and subsequent accumulation of extracellular matrix (ECM) in CKD, and MC hypertrophy has been recognized as the fundamental change in the pathogenesis [10, 11]. Renal cells are a rich source of TGF-*β*1. Diverse factors such as high glucose, Ang II, and AGE could stimulate the production of TGF-*β*1 in MC [12–14]; treatment of diabetic mice with a neutralizing anti-TGF-*β* antibody could completely prevent diabetic glomerular hypertrophy [15]. TGF-*β*1 induced MC hypertrophy may be mediated by PKC-independent mechanisms [16], regulation of IP3Rs [15], AGE-RAGE-mediated reactive oxygen species (ROS) generation [14], a feedback loop between deptor downregulation and TORC1/2 activation instilled by Smad3 [17], or PI3K-Akt activation through FOG2 downregulation by miR-200b/c [18]. Evidences are emerging from* in vivo* and* in vitro* studies that p21 is required for MC or glomerular hypertrophy induced by TGF-*β*1 [5, 9, 19–22], but how TGF-*β*1 regulates p21 is not fully clear.

Epigenetic modifications have been increasingly recognized as novel, important contributors to gene regulation in multiple diseases. Epigenetic mechanisms include DNA methylation, posttranslational modification (PTM), and miRNA regulation. The most abundant PTMs are the phosphorylation, acetylation, methylation, and ubiquitylation at amino-terminal tails of lysines on histone H3 or H4. Gene activation is usually associated with lysine acetylation on H3 and H4 residues (HKAc), methylation of H3 lysine 4 (H3K4me), trimethylation of H3 lysine 79 (H3K79me3), and trimethylation of H3 lysine 36 (H3K36me3), whereas di- or trimethylation of H3 lysine 9 (H3K9me2/3) and trimethylation of H3 lysine 27 (H3K27me3) are linked to gene repression; there is also crosstalk between active and repressive modifications in gene regulation. The enhanced level of TGF-*β*1-mediated p21 mRNA in RMCs has been reported to be associated with H3K9/14Ac levels [22], and TGF-*β*1 induced ECM associated genes upregulation was associated with enhanced levels of H3K4me1/2/3 and decreased H3K9me2/3 levels [23]; there is no report on the role of histone methylation in TGF-*β*1-induced p21 gene expression in RMC. SET7/9 has been identified as a histone lysine methyltransferase (HMT), generating monomethylation of histone H3 lysine 4 (H3K4me1); several reports have found that SET7/9 and the associated H3K4me1 involved in profibrotic and inflammatory genes regulation [23–26]. TGF-*β*1 stimulation upregulated SET7/9 expression and SET7/9 recruitment at ECM associated genes promoters, and knockdown of SET7/9 with siRNAs could partly abolish TGF-*β*1 induced ECM associated genes upregulation. However, the roles of active and repressive histone H3 lysine methylation (H3Kme) and key HMT SET7/9 in the regulation of TGF-*β*1 mediated p21 gene expression in RMCs are not clear.

In this report, we demonstrate that TGF-*β*1 induces decreased levels of H3K9me and the enrichment of H3K4me at p21 promoter in RMCs, at the same time we elucidate that HMT SET7/9 plays a key role in TGF-*β*1 mediated p21 expression. These data demonstrate a novel epigenetic mechanism of TGF-*β*1 induced p21 gene expression leading to MC hypertrophy, the characteristic of chronic renal diseases.

## 2. Methods

### 2.1. Cell Culture and Stimulation

Primary RMCs were obtained by explant culture of renal glomeruli isolated from Sprague-Dawley male rats (160–180 g) as described [23] with the allowance of the Ethics Committee on the Care and Use of Laboratory Animals of the Second Hospital of Jilin University (Changchun, China). RMCs were maintained in RPMI 1640 medium. The subsequent experiments were performed with RMCs from passages 6–10. RMCs were serum depleted in 0.2% BSA medium for 24 h prior to stimulation. RMCs were treated with 10 ng/mL Recombinant Human TGF-*β*1 (240-B, R&D systems, Minneapolis, MN) as indicated time, while control groups were treated with the vehicle.

### 2.2. Transfections of SET7/9 Small Interfering RNA (siRNA) into RMC

SET7/9 ON-TARGETplus siRNA (J-059399(09-12))(siSET7/9) was from Thermo Scientific; and Silencer® Negative Control #1 siRNA (AM4611)(siNeg) was from Ambion, Inc. Transfection of siRNAs to RMC was performed as described previously [23]. About 24 h after transfection, RMCs were depleted with serum-free RPMI 1640 medium containing 0.2% BSA for 24 h, then stimulated in the presence or absence of TGF-*β*1 (10 ng/mL) for 6 h, and processed the detection for RNA levels by RT-QPCR.

### 2.3. RNA Isolation and RT-QPCR Analysis

Total RNA was extracted from RMCs using TRIzol reagent (Invitrogen, Carlsbad, CA, USA) according to the manufacturer's instructions; the RNA concentration was determined with the sample dissolved in diethylpyrocarbonate-treated water. Isolated RNA (2 *μ*g) was used in reverse transcription using* Tag*Man Reverse Transcription Kit (Applied Biosystems Inc., Foster City, CA, USA) according to the manufacturer's protocol. The synthesized cDNA (3 *μ*L) was used for quantitative real-time PCR (QPCR) amplication. The specific primers were synthesized by Shanghai Invitrogen Biotechnology Co., Ltd. (listed in Table 1). QPCRs were performed with SYBR-green reagent (Life technologies, UK) in triplicate in a final volume of 20 *μ*L with ABI 7300 real-time PCR thermal cycler. Dissociation curves were run to detect nonspecific amplification and confirm single product was amplified in each reaction. The comparative cycle time (Ct) method was applied to determine fold differences between samples, with the values normalized with internal control *β*-actin gene by 2^−ΔΔCt^ method.

### 2.4. Western Blot Analysis

Proteins were extracted from RMCs samples and were homogenized in RIPA buffer (150 mM NaCl, 50 mM Tris-HCl, 1% NP-40, 0.5% sodium deoxycholate, and 0.1% SDS) plus protease and phosphatase inhibitors mixture. Equal amounts of protein samples (30 *μ*g) were separated by 10% SDS-PAGE gels and transferred to PVDF membranes. The membranes were blocked with 5% milk for 1 h at room temperature. The immunoblotting was performed with primary antibody against p21 (MA1-91045, Thermo; 1 : 1000) and incubated overnight at 4°C. Proteins were detected with Chemiluminescence method. The blots were stripped and then reprobed with an antibody against *β*-actin (A5441, Sigma Aldrich, St. Louis, MO; 1 : 100000) as a load control. The densitometric analysis was performed using ImagJ (Wayne Rasband, National Institutes of Health, Bethesda, MD).

### 2.5. Chromatin Immunoprecipitation (ChIP) Assays

RMCs were treated with TGF-*β*1 for 24 h and then cross-linked with 1% formaldehyde for 10 min at 37°C, washed twice with cold PBS supplemented with protease inhibitors, and lysed as described previously [23]. Cell lysates were sonicated and diluted. Immunoprecipitation was performed overnight at 4°C with antibodies to methylated histones (H3K4me1 (ab8895), H3K4me2 (ab32356), H3K4me3 (ab8580), H3K9me2 (ab1220), and H3K9me3 (ab8898); Abcam, Cambridge, MA), SET7/9 (11209, Sino Biological Inc), or IgG (antibody control). Immune complexes were obtained using protein A/G beads and washed to remove nonspecific binding; bound proteins were eluted and ChIP-enriched DNA was collected by phenol and chloroform extraction. Antibody-enriched ChIP DNA and input DNA samples were analyzed by real-time PCR with indicated primers within p21 and control cyclophilin A (CypA) promoters (See Figure 2(a) and Table 1). All reactions were carried out in triplicate in a final volume of 20 *μ*L. Data were analyzed with the 2^−ΔΔ*Ct*^ method and normalized to input samples. Results were expressed as fold over control.

### 2.6. Statistical Analysis

Values were expressed as mean ± SEM of multiple experiments. Differences between groups were assessed by Paired Student's *t*-tests or ANOVA with Dunnet's posttests. Statistical tests were performed using the GraphPad Prism 5.0 software. *P* < 0.05 was considered to be statistically significance.

## 3. Results

### 3.1. TGF-*β*1 Increases p21 Gene Expression, While Reciprocally Inhibitory H3K9me Levels Are Downregulated at Its Promoter in RMC

**Figure 1:**
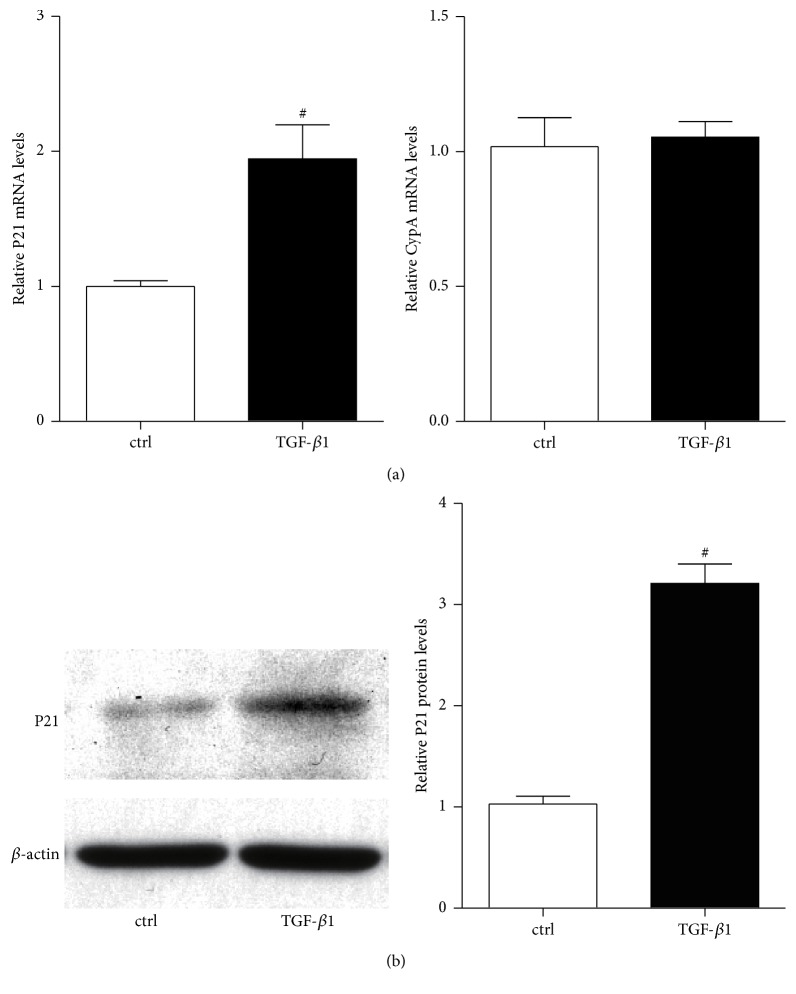
Changes in relative expression of the p21 mRNA and protein between TGF-*β*1 treatment and control (ctrl) groups. TGF-*β*1 treated MC and control groups are investigated by RT-QPCR (a) and western blot (b); *β*-actin was used as internal control gene. Results are expressed as fold over control (mean ± SEM; ^#^ *P* < 0.05 versus ctrl, *n* = 3).

Firstly we examined p21 gene expression in RMC in the presence or absence of TGF-*β*1 (10 ng/mL) for different time through RT-QPCR and western blot, and whether TGF-*β*1 induced p21 expression was associated with the changes in the repressive epigenetic marks H3K9me2 and H3K9me3 at its promoter. Serum depleted RMC was treated with or without TGF-*β*1 for 6 h, and p21 gene expression level was determined by RT-QPCR. p21 mRNA level was significantly increased in the presence of TGF-*β*1 compared with control, while there was no difference in the housekeeping gene CypA between the two groups (Figure 1(a)). Western blot analysis showed that p21 protein level (Figure 1(b)) was also similarly increased in the presence of TGF-*β*1 for 24 h. These results suggested that TGF-*β*1 can increase the expression of p21 gene in RMC as expected.

**Figure 2:**
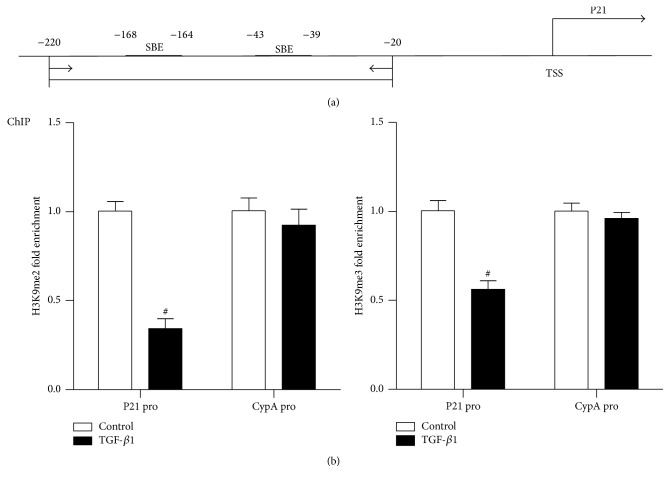
TGF-*β*1 downregulates H3K9me2/3 levels at p21 gene promoter in RMC. (a) Map showing locations of p21 promoter primer used for ChIP-QPCRs. TSS: transcription start site; SBE: Smad binding elements. (b) ChIP assays performed with H3K9me2 and H3K9me3 antibodies normalized to input DNA. Results are expressed as fold over control (mean ± SEM; ^#^ *P* < 0.05 versus ctrl, *n* = 3).

Next we examined the methylation status of H3K9 using ChIP assays with specific antibodies of H3K9me2 and H3K9me3. ChIP-enriched DNA samples were analyzed by QPCR using primers spanning Smad binding element (SBE) in the proximal region of the p21 promoter (Figure 2(a)). Levels of both H3K9me2 and H3K9me3 (Figure 2(b)) at p21 promoter were significantly decreased in RMC in the presence of TGF-*β*1 for 24 h compared with the absence of TGF-*β*1 (control, ctrl). In contrast, there was no significant difference at CypA promoter between two groups. These results suggested that TGF-*β*1-induced p21 expression may be due, at least partly, to the loss of inhibitory histone lysine methylation at its promoter.

### 3.2. TGF-*β*1 Increases H3K4me Levels at the p21 Promoter

**Figure 3:**
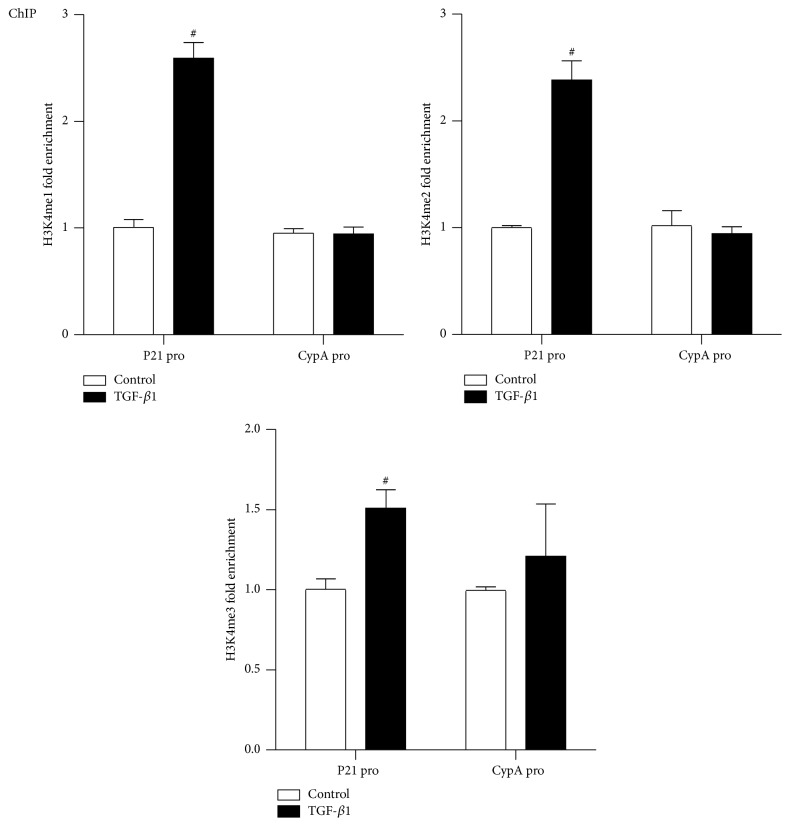
TGF-*β*1 increases H3K4me1/2/3 levels at p21 gene promoter in RMC. ChIP assays performed with H3K4me1, H3K4me2, and H3K4me3 antibodies normalized to input DNA. Results are expressed as fold over control (mean ± SEM; ^#^ *P* < 0.05 versus ctrl, *n* = 3).

It has been recognized that specific posttranslational modifications of histones such as acetylation or methylation were associated with the activation of specific genes. We next investigated whether TGF-*β*1 could influence H3K4me levels at the gene promoter of p21, the epigenetic “active” marks, using ChIP assays with H3K4me1, H3K4me2, and H3K4me3 antibodies. As shown in Figure 3, 24 h TGF-*β*1 treatment significantly enhanced H3K4me1, H3K4me2, and H3K4me3 levels at the p21 promoter in RMC to different extent, and these increases in H3K4me1/2/3 levels at p21 promoter are consistent with the upregulated expression of p21 gene after TGF-*β*1 stimulation. On the other hand, there were no significant differences in these marks at the CypA promoter, confirming the specificity. These results indicated that increased H3K4 methylation levels might be involved in TGF-*β*1 induced p21 gene upregulation in RMC.

### 3.3. TGF-*β*1 Increases SET7/9 Recruitment to p21 Gene Promoter

**Figure 4:**
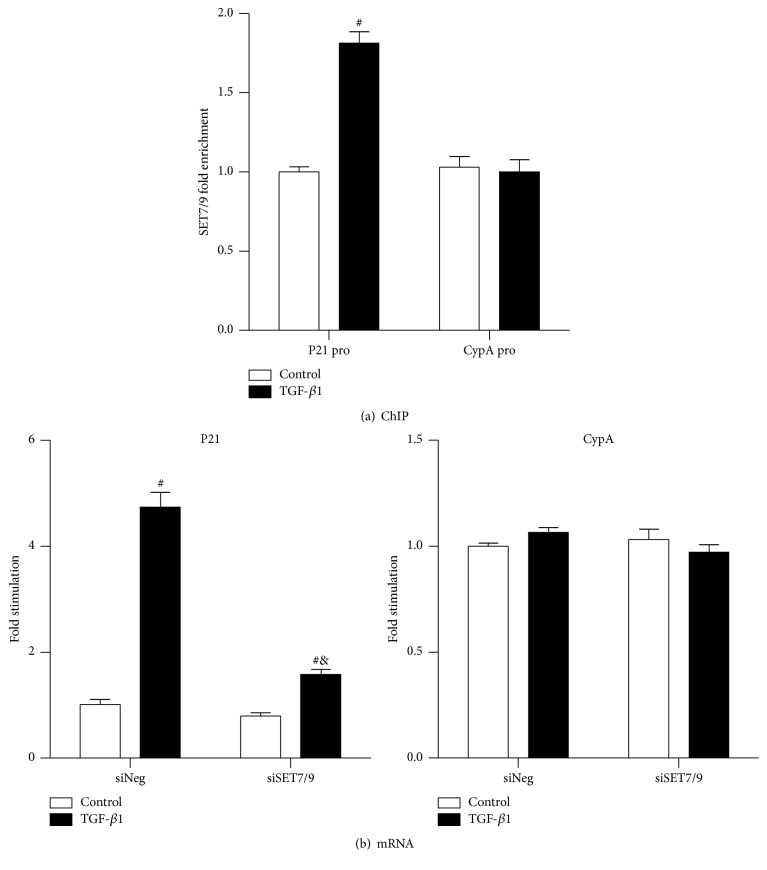
TGF-*β*1 enhances SET7/9 recruitment at p21 gene promoter and SET7/9 is involved in TGF-*β*1 induced p21 gene expression in RMC. (a) ChIP assays performed with SET7/9 antibody normalized to input DNA. Result was expressed as fold over control (mean ± SEM; ^#^ *P* < 0.05 versus ctrl, *n* = 3). (b) p21 mRNA expressions are investigated by RT-QPCR; *β*-actin was used as internal control gene. Results are expressed as fold over control (mean ± SEM; ^#^ *P* < 0.05 versus ctrl; ^&^ *P* < 0.05 versus siNeg + TGF-*β*1, *n* = 3).

The HMT SET7/9 has been well characterized in gene regulation [23, 25–28]. In previous study, TGF-*β*1 has been proven to be a contributor to increase SET7/9 gene expression as well as SET7/9 occupancy to the promoters of ECM associated genes (Col1*α*1, CTGF, and PAI-1) [23]. Here, we investigated whether TGF-*β*1 alters SET7/9 occupancy on the p21 promoter using ChIP assays with SET7/9 antibody. As shown in Figure 4(a), SET7/9 recruitment was significantly increased at p21 promoter in the presence of TGF-*β*1 for 24 h compared with TGF-*β*1 absent group (ctrl), and there was no significant difference at the CypA promoter in the TGF-*β*1 presence and absence groups. The SET7/9 recruitment pattern suggested an association with the increased H3K4me1 level induced by TGF-*β*1 (Figure 3), indicating a key function of SET7/9 in TGF-*β*1-mediated upregulation of H3K4me1 level in the induction of the p21 gene.

### 3.4. Knockdown of SET7/9 Partly Abolished TGF-*β*1-Induced p21 Gene Expression

In the previous study we have shown that knockdown of SET7/9 by siSET7/9 could partly abolish TGF-*β*1-induced ECM associated genes expression. To better understand the role of SET7/9 in TGF-*β*1 induced p21 gene expression, RMCs were transfected with 300 ng siRNA oligonucleotides targeting SET7/9 (siSET7/9) or negative control siRNAs (siNeg) and then treated with or without TGF-*β*1 (10 ng/mL) for 6 h, and mRNA levels were analyzed by RT-QPCR. As shown in Figure 4(b), TGF-*β*1-induced p21 mRNA level was significantly attenuated in siSET7/9 transfection group compared with siNeg group. In contrast, there were no significant changes in CypA mRNA levels induced with or without TGF-*β*1 between siSET7/9 and siNeg groups (Figure 4(b)). These findings further supported the key role of SET7/9 in regulating TGF-*β*1-induced p21 gene expression in RMC.

## 4. Discussion

In the present report, firstly we confirmed that TGF-*β*1 treatment could upregulate p21 gene expression in RMC; then we found significant changes of histone lysine methylations, including H3K9me2/3 and H3K4me1/2/3 levels at p21 gene promoter, which were closely associated with p21 gene expression; further results showed that SET7/9, the specific H3K4me1 HMT, was involved in TGF-*β*1 induced p21 gene upregulation in RMC.

It is widely known that CDKI p21^Cip1/WAF1^ (p21) plays a key role in MC hypertrophy and glomerular hypertrophy leading to chronic renal diseases including DN [1, 3, 4, 7, 8, 29, 30]; p21 is the target gene of multiple cytokines and growth factors including TGF-*β*1 [5, 8, 19]. However, p21 expression can be modulated through PTM mechanisms [29, 31]. Multiple studies have revealed that the key role of histone PTMs, such as phosphorylation, acetylation, and methylaiton, in gene transcription [32, 33]. For instance, TGF-*β*1 induced H3K9/14Ac and acetylation of Smad2/3 playing key roles in p21 gene expression in MC. Howerver, to the best of our knowledge, there is no data about histone lysine methylation and SET7/9 associated mechanism in modulating p21 gene expression.

H3K9me2 and H3K9me3 have been extensively studied and characterized as contributors to transcriptional repression and gene silencing in general [23, 34–36]. In a previous experiment using cultured vascular smooth muscle cells (VSMCs) from diabetic db/db mice, under normal conditions and exposed to TNF-*α* conditions, led to decreased levels of H3K9me3 at key inflammatory genes promoters and inversely correlated with the increased expression of these inflammatory genes [37]. Further study in cultured human VSMCs and endothelial cells showed that exposure to HG also led to decreased H3K9me3 levels, implicating that downregulation of repressive H3K9me3 can increase proinflammatory genes expression under diabetic conditions [37, 38]. In addition, data from our previous study suggested that H3K9me2 and H3K9me3 levels were decreased at ECM associated genes promoters in TGF-*β*1 and HG stimulated RMCs, which were inversely correlated with the increased expression of these profibrotic genes [23]. Similarly to these collective findings, our current results demonstrated that TGF-*β*1 decreased H3K9me2 and H3K9me3 levels in the promoter of p21 for the first time, which ultimately led to p21 gene upregulation, suggesting that decrease of repressive histone lysine methylation may involve in TGF-*β*1 induced p21 gene expression.

Increasing evidences showed that histone H3 lysine 4 methylation (H3K4me) including mono-, di-, and trimethylation enriched at promoter, enhancer, and other regulatory sequences is broadly associated with active genes expression [34, 39–42]. Our previous results showed that TGF-*β*1 induced H3K4me1/2/3 increases in parallel with the decreases of H3K9me2/3 at ECM associated genes promoters, suggesting that H3Kme changes may further cause these genes upregulation [23]. To further understand the key roles of H3K4me in regulating TGF-*β*1-induced p21 expression, we investigated the levels of H3K4me1/2/3 in RMC exposed to TGF-*β*1 for 24 h; subsequently our current results showed that TGF-*β*1 upregulated p21 gene expression in RMC, which is positively correlative with the increased H3K4me1/2/3 levels at the p21 promoter.

HMT STE7/9 has been demonstrated to not only specially catalyze H3K4me1 [26, 43], but also methylate nonhistone proteins such as P53 [44, 45], TAF10 [46], DNMT1 [47], and P65 [48, 49]. SET7/9 was reported to regulate the expression of HG-induced NF-*κ*B subset p65 and inflammatory genes expressions in endothelial cells, which were involved in the “metabolic memory” phenomenon [24]. Similarly, SET7/9 took part in the TNF-*α*-induced target inflammatory genes expressions of NF-*κ*B pathway in monocytes [25]. A previous study showed that TGF-*β*1 could increase both the expression of SET7/9 and the recruitment of SET7/9 at the ECM associated genes promoters, and knockdown of SET7/9 could specially decrease global H3K4me1 level; all the data suggested that SET7/9-mediated H3K4me1 was involved in the TGF-*β*1-induced ECM genes upregulation in RMCs. Similarly, in the present study we demonstrated that TGF-*β*1 could increase SET7/9 occupancy at p21 gene promoter, and silencing SET7/9 with siRNAs could partly abolish TGF-*β*1-induced p21 gene upregulation in RMC, supporting that SET7/9-dependent H3K4me1 plays a key role in p21 expression and SET7/9 could be a strong preventive agent against RMC hypertrophy in CKD. Further studies are necessary to reveal cooperative roles of other HMTs mediating H3K4me2/3 and H3K9me2/3 in modulating p21 gene expression in response to TGF-*β*1.

In a recent study in MC linked to DN, TGF-*β*1 has been shown to upregulate H3K9/14Ac level at p21 promoter and increase p21 gene expression mediated by HATs including CBP and p300, but not p/CAF [22], and in the present study we demonstrate that TGF-*β*1 can decrease the levels of repressive chromatin histone methylation marks H3K9me in RMC, which are associated with the upregulation of p21 expression, suggesting that a cooperative role between active and repressive marks at the K9 site and a crosstalk between HMTs and HATs may control TGF-*β*1-induced p21 expression. Several previous studies have revealed that HDACs are involved in the production of TGF-*β*1 induced ECM and subsequent kidney fibrosis associated with diabetic kidneys [50, 51]. Further studies are needed to demonstrate such interplay between histone lysine methylation and acetylation and the roles of respective catalytic enzymes in gene expression.

In conclusion, our study provides extensive evidence that TGF-*β*1-induced p21 gene expression related to MC hypertrophy is correlative with the significant changes in promoter histone H3K9 and H3K4 methylations in RMCs; HMT SET7/9 plays an important role in the pathogenesis. Therefore, it is possible that histone lysine methylations and HMT SET7/9 could serve as potential therapeutic agents for MC hypertrophy associated CKD including DN.

## Figures and Tables

**Table 1:** Sequences of RT-QPCR and ChIP-QPCR primers.

Primer	Forward primer	Reverse primer	Annealing temprature
cDNA primers			
rp21	TGTTCCACACAGGAGCAAAG	AACACGCTCCCAGACGTAGT	58
rCypA	TATCTGCACTGCCAAGACTGAGTG	CTTCTTGCTGGTCTTGCCATTCC	58
r*β*-actin	CTGCCCTGGCTCCTAGCAC	cggacGCAGCTCAGTAACAGTCcG	62
ChIP primers			
rp21pro	CGCCCCTTTCTAGCTGTCTG	CAGATCTGCGGTCTTATAGCATC	58
rCypApro	TATCTGCACTGCCAAGACTGAGTG	CTTCTTGCTGGTCTTGCCATTCC	58
